# Knowledge, attitudes and practices of primary care professionals about the food guide for children up to 2 years old: a cross-sectional study, Botucatu, São Paulo, Brazil, 2023

**DOI:** 10.1590/S2237-96222024v33e20240111.en

**Published:** 2024-12-13

**Authors:** Vivian Lovison do Amaral, Giovana Canela Spadotto, Caroline de Barros Gomes

**Affiliations:** 1 Hospital das Clínicas da Faculdade de Medicina de Botucatu, Especialização em Redes de Atenção no Sistema Único de Saúde, Botucatu, SP, Brazil; 2 Universidade Estadual Paulista, Faculdade de Medicina de Botucatu, Programa de Pós-Graduação em Saúde Coletiva, Botucatu, SP, Brazil

**Keywords:** Breastfeeding, Infant Nutrition, Food Guides, Primary Health Care, Cross-Sectional Studies, Lactancia Materna, Nutrición del Lactante, Guías Alimentarias, Atención Primaria de Salud, Estudios transversales

## Abstract

**Objective:**

To investigate knowledge, attitudes and practices of primary health care professionals regarding breastfeeding and complementary feeding, in accordance with the recommendations of the food guide for Brazilian children up to 2 years old.

**Methods:**

This is a descriptive study undertaken from October to December 2023, with a self-administered online questionnaire, aimed at physicians, nurses and community health agents in primary health care in Botucatu, São Paulo, Brazil. We performed descriptive analysis and used Pearson’s chi-squared test to analyze association between professional categories and their knowledge, attitudes and practices in relation to the content of the food guide.

**Results:**

74 professionals participated, including 37 community health agents, 19 nurses and 18 physician. Half the professionals had low knowledge about the content of the guide, getting less than half the answers right, in particular those related to complementary foods, for which 57 professionals got less than half of them right. Physicians and nurses were the professionals who believed they were more qualified to provide information related to the content of the guide, when compared to community health agents (p-value<0.001). Regarding practices, 18 professionals stated they sometimes provide guidance on breastfeeding, 24 reported never doing breastfeeding assessments during consultations and home visits and 22 provided guidance on introduction of complementary feeding.

**Conclusion:**

Knowledge of physician, nurses and community health workers was deficient regarding the content of the guide. Attitudes and practices regarding the content were also compromised.

## Resumen

**Objetivo:**

Investigar conocimientos, actitudes y prácticas de los profesionales de la atención primaria de salud sobre la lactancia materna y la alimentación complementaria, de acuerdo con las recomendaciones de la guía alimentaria para niños brasileños menores de 2 años.

**Métodos:**

Se trata de un estudio descriptivo realizado de octubre a diciembre de 2023, con cuestionario autoadministrado en línea, dirigido a médicos, enfermeros y agentes comunitarios de salud de la atención primaria de salud en Botucatu, São Paulo, Brasil. Se realizó un análisis descriptivo y de asociación entre categorías profesionales y sus conocimientos, actitudes y prácticas en relación al contenido de la guía, mediante la prueba chi-cuadrado de Pearson.

**Resultados:**

Participaron 74 profesionales, entre ellos 37 agentes comunitarios de salud, 19 enfermeros y 18 médicos. La mitad de los profesionales tenía conocimientos bajos sobre el contenido de la guía, acertando menos de la mitad de las preguntas, con especial énfasis en las relacionadas con alimentos complementarios, en las que 57 profesionales acertaron menos de la mitad de las mismas. Los médicos y enfermeros fueron los profesionales que más se creyeron más capacitados para transmitir informaciones relacionadas al contenido de la guía, en comparación con los agentes comunitarios de salud (p<0,001). En cuanto a las prácticas, 18 profesionales afirmaron brindar en ocasiones orientaciones sobre la lactancia materna, 24 afirmaron no realizar nunca evaluaciones de la lactancia materna durante las consultas y visitas domiciliarias y 22 de ellos brindaron orientaciones sobre la introducción de la alimentación complementaria.

**Conclusión:**

El conocimiento de médicos, enfermeras y trabajadores comunitarios de salud fue deficiente respecto al contenido de la guía. Las actitudes y prácticas respecto de dichos contenidos también se han visto comprometidas.

**Ethical aspects**

**This research respected ethical principles, having obtained the following approval data**

Research ethics committee: Faculdade de Medicina de Botucatu

Opinion number: 6.463.135

Approval date: 26/10/2023

Certificate of submission for ethical appraisal: 74345323.2.0000.5411

Consent form: Obtained from all participants before data collection

## Introduction

Feeding in the first two years of life plays an essential role in growth, development, habit formation and health. Inadequate eating practices increase the risk of malnutrition and chronic diseases, considering that the nutritional status of children under two years of age is directly influenced by these practices ^1^.

The first food to be given to a baby is breast milk. The benefits of this, especially exclusive breastfeeding until the sixth month and continued breastfeeding until 2 years or more, involve the mother and continue into adulthood. Benefits include reduced risk of ovarian and breast cancer, lower rates of diarrhea and respiratory diseases in the first two years of life and lower rates of obesity in childhood and adulthood ^2^. After the period of exclusive breastfeeding, in the first 180 days of life, appropriate complementary feeding is related to better eating habits for life and lower rates of food allergies and excess weight ^3^ ^,^ ^4^.

Taking care of nutrition in this age group (0-23 months) is essential for proper development and health promotion ^5^. Guidance given by healthcare professionals must be updated and based on scientific evidence, as inadequate conduct during this period can result in harm to health. ^6^.

Primary health care is the main field for promoting adequate nutrition in the first two years of life, being the service user’s gateway into the Brazilian National Health System (*Sistema Único de Saúde* - SUS), coordinating care. Care related to food and nutrition at this level of assistance includes promotion of adequate and healthy eating at all stages of life. This diet should be encouraged by all health professionals, respecting their core competencies ^7^.

The food guide for Brazilian children up to 2 years old, published in 2019 by the Ministry of Health, offers guidelines on breastfeeding and complementary feeding. This guide follows the World Health Organization recommendation that governments develop national guidelines to support families and guide public policies ^1^. 

In Currais Novos, in the state of Rio Grande do Norte, it was found that a high percentage of primary health care professionals were unaware and/or had doubts about breastfeeding and complementary feeding ^8^. In six health centers in Teresina, in the state of Piauí, health professionals had adequate knowledge about exclusive breastfeeding, however they needed to improve their guidance on complementary feeding ^9^. It is noteworthy that both circumstances refer to breastfeeding and complementary feeding, and not specifically to the food guide.

It is important for health professionals to be aware of the recommendations contained in the guide, and that this knowledge, as well as its application, be investigated. The objective of this study was to investigate the knowledge, attitudes and practices of primary health care professionals in Botucatu, São Paulo, about breastfeeding and complementary feeding, in accordance with the recommendations of the food guide for Brazilian children up to 2 years old.

## Methods

### Study design and population

This is a descriptive study carried out in Botucatu, a municipality in the Center-South of the state of São Paulo, Brazil. In 2022, Botucatu had an estimated population of 145,155 inhabitants. In 2020, the infant mortality rate was 10.94 deaths per 1,000 live births. The latest Human Development Index data (0.800), dating from 2010, was higher than the national average ^10^.

Botucatu’s primary care network was made up of 22 health centers at the time, divided into three different service models: two teaching health centers, six primary health centers and 14 family hHealth strategy centers ^11^.

All physicians, nurses and community health agents over 18 years old who worked in any of the 22 primary care network centers in the city were considered eligible and invited to take part in the study. Considering a certain fluctuation in the number of professionals, at the time the municipality had approximately 99 community health agents, 72 nurses and 85 physicians (unpublished data). 

### Data collection and variables

Data collection took place between October and December 2023, using a non-validated online form, developed by the authors. The invitation to health professionals to participate in the study was made via the coordinator of each health center, after the researchers had contacted the municipal health department management and the social organization that manages part of the centers. The team carried out in-person visits, reinforcing the invitation with the managers of the centers, and distributed reminders with the questionnaire QR code to eligible professionals. Two weeks after the visit, a new reminder was sent via messaging application to the managers of the centers.

Before starting the questionnaire, each professional accepted the Informed Consent Form for the study. The questionnaire included socio-professional characterization questions, followed by knowledge, attitudes and practices in relation to breastfeeding and complementary feeding. All these questions covered the content of the food guide for Brazilian children up to 2 years old. 

The items related to the characterization of professional profile and job were biological sex, age, race/skin color (White, Black, mixed race, Asian, other), profession (community health agent, physician or nurse), health center to which they belonged, length of service in primary care, schooling and academic and professional training (undergraduate, specialization, residency, master’s degree, doctorate). The next section of the questionnaire addressed whether the professional knew the food guide (yes, no), whether they had already participated in any training on the guide (yes, no), training on breastfeeding (yes, no) and training on complementary feeding (yes, no), and how long ago they had completed this training. 

The sections on knowledge and attitudes regarding breastfeeding and complementary feeding included questions on a Likert scale, in which participants expressed their level of agreement through five statements that ranged from “strongly disagree” to “strongly agree”. In the section on practices, the questions involved the alternatives “never”, “rarely”, “sometimes”, “often” and “always”. The data obtained were imported into an Excel spreadsheet, which was used to build the database. All questions were mandatory.

### Statistical analysis

Descriptive analyses were performed on the variables investigated, including calculation of absolute and relative frequencies for categorical and mean variables, 95% confidence intervals and minimum and maximum values for continuous variables. Association between the different professional categories and the results found regarding knowledge of the guide, their attitudes and practices in relation to the recommended content regarding breastfeeding and the introduction of complementary foods was investigated using Pearson’s chi-squared test. 

When assessing knowledge, the professionals’ answers were categorized as right or wrong, considering the alternative “strongly agree” or “strongly disagree” as the right answer, depending on the question asked, organizing this variable as a continuous one. The number of right answers was added separately for breastfeeding (maximum score=19) and for complementary feeding (maximum score=18), and stratified into three categories: ≥15 right answers; 9-14 right answers; ≤8 right answers. Association between the right answer category and professional category was investigated using Pearson’s chi-squared test. All the analyses were performed using the Statistical Package for Social Sciences for Windows, version 20.0, considering a p-value<0.05 as the level of statistical significance.

## Results

A total of 74 health professionals participated in the research, including 37 community health agents, 19 nurses and 18 physicians, which makes up approximately 37% of community health agents, 26% of nurses and 21% of physicians who work in municipal primary care health services in Botucatu. With regard to graduation, 18 professionals had graduated less than five years ago, and 31 of them reported having a specialization course or medical residency. Regarding the health service center model and length of experience, 53/74 worked in a family health strategy center model and 40 reported that they had been working for less than five years in primary health care (Table 1).

**Table 1:** Characterization of physicians, nurses and community health agents working in primary health care and participating in the study. Botucatu, São Paulo, Brazil, 2023 (n=74)

Characteristics	n
**Age (years)**	
18-25	10
26-29	6
30-39	26
40-49	22
50-59	7
>60	3
**Sex**	
Female	61
Male	13
**Race/skin color**	
White	62
Mixed race	11
Black	1
**Characteristics**	**n**
**Schooling**	
Complete high school education	14
Complete higher education	20
Incomplete higher education	10
Postgraduate education	30
**Profession**	
Community health agent	37
Nurse	19
Physician	18
**Time since graduated (years)a**	
<5	18
5-9	8
10-14	6
15-20	8
>20	10
**Specialization/residencya**	
Yes	31
No	19
**Masters degreea**	
Currently taking	2
Concluded	10
Has not taken	38
Doctoratea	
Currently taking	1
Concluded	6
Has not taken	43
**Type of heath service worked in**	
Family Health Strategy	53
Primary Health Center	7
Teaching Health Center	10
Polyclinic	4
**Length of service in primary health care (years)**	
<5	40
5-9	7
10-15	6
15-20	11
>20	10

Note: a24 data missing due to not having taken a degree course.

Only 11 professionals had knowledge of the guide, while 19/74 did had no knowledge of it. A statistical difference was found between the professional categories, with physicians and nurses being the professionals who most had knowledge of the guide when compared to community health agents (p-value 0.009). The majority (n=46) strongly disagreed that the guide is a Ministry of Health publication aimed only at nutritionists, and 34/74 strongly agreed that it can be used by families who have babies in this age group, with no statistical difference between professional categories (p-value 0.414 and p-value 0.241). Of the total number of participating professionals, 69 had no training regarding the guide, 42 reported having had training on breastfeeding and 52 had not had training on complementary feeding, with no statistical difference between professional categories (data not shown).

The majority of professionals (64/74) strongly agreed that breast milk by itself is enough for a baby in the first six months of life, and 69 of them strongly disagreed that small breasts produce little milk. Only 25/74 strongly agreed that one of the breastfeeding positions is lying down, next to the baby. Issues such as the consumption of hominy to increase milk production, breastfeeding beyond 2 years and the child’s independence and the same issue of breastfeeding lying down were those which the professionals were the most uncertain about, revealed by higher percentages between somewhat agreeing and somewhat disagreeing (Table 2).

**Table 2:** Knowledge of physicians, nurses and community health agents on the breastfeeding content of the food guide for Brazilian children under 2 years old. Botucatu, São Paulo, Brazil, 2023 (n=74)

Questions	Strongly agree	Somewhat agree	Neither agree or disagree	Somewhat disagree	Strongly disagree
Breast milk can be weak	1	2	2	8	61
Small breasts produce little milk	0	1	0	4	69
Hominy is an example of a food that helps increase breast milk production	5	10	24	9	26
Mothers with flat or inverted nipples can breastfeed	48	15	5	3	3
It is recommended to alternate breasts at each feeding	41	16	4	5	8
Mothers infected with HIV can breastfeed	13	8	6	1	46
Breastfeeding is recommended in the first hour of life	64	5	4	0	1
Breastfeeding should occur whenever the child wants, without predefined times or intervals	43	17	3	7	4
Breast milk by itself is enough for a baby in the first 6 months of life	64	8	0	0	2
Breastfeeding must be exclusive until 6 months (no other milk, formula, water, tea, juices or foods)	56	10	2	3	3
Breastfeeding beyond 2 years harms the child’s natural process of becoming independent	8	12	12	15	27
Children who breastfeed have more difficulty accepting other foods	1	5	4	15	49
One of the positions for the mother to breastfeed is lying down, next to the baby	25	15	5	12	17
Mothers with engorged (“swollen”) breasts should be advised to apply a warm compress to their breasts	16	17	6	9	26
Pacifiers can cause nipple confusion and interfere with breastfeeding	46	14	1	6	7
Feeding bottles can cause nipple confusion and interfere with breastfeeding	49	15	3	4	3
Lactating women (women who breastfeed) who become pregnant should stop breastfeeding	12	9	8	12	33
On very hot days, even children who are exclusively breastfed should be given water	8	4	10	13	39
Even with the introduction of new foods, breastfeeding should be continued for up to 2 years or more	37	27	4	3	3

The majority (55/74) of professionals strongly agreed that the recommended age for starting food introduction is at 6 months old and that ultra-processed foods should not be given to children until they are 2 years old (57/74; Table 3). Only 8 professionals strongly agreed that foods with allergenic potential such as eggs, fish, soy and seafood can be given from the beginning of food introduction. More than half stated that fruit juice can be given from the beginning of food introduction (40/74). Many doubts were found in relation to the other questions asked, with high percentages in the intermediate alternatives (somewhat agree, neither agree or disagree, somewhat disagree).

**Table 3:** Knowledge of physicians, nurses and community health agents about the complementary feeding content of the food guide for Brazilian children under 2 years old. Botucatu, São Paulo, Brazil, 2023 (n=74)

Questions	Strongly agree	Somewhat agree	Neither agree or disagree	Somewhat disagree	Strongly disagree
The recommended age for starting food introduction is at six months old	55	14	3	1	1
One of the signs of readiness to start complementary feeding is sitting without any or little support.	24	16	15	6	13
Children who are not breastfed and whose family does not have money to buy infant formula can be fed modified cow's milk up to 4 months old	10	13	19	13	19
Food, at the beginning of food introduction, must be blended in a blender or mixer or sieved before being given to babies	19	8	7	7	33
Garlic, onion and chives can be included to season your baby’s food from the first day it starts eating	22	17	11	4	20
Food introduction should begin with fruit and only after good acceptance of fruit, introduce baby food/ savory food	22	23	6	8	15
Leguminous food (beans, lentils, chickpeas, peas) should be part of the meal from the beginning of the introduction of complementary foods for the baby	28	17	9	11	9
At the beginning of food introduction, it is not recommended to give pork and eggs, even if well cooked	29	17	6	9	13
For main meals (lunch and dinner), the plate must contain one item from each food group: beans; cereals or tubers or roots; meat or eggs; vegetables and greens	46	13	10	3	2
Fruit juices (without sugar, only with fruit) can be given from the beginning of food introduction	40	10	3	7	14
Sugar, added to food such as cakes, juices and pies, can be given to children from the first year of life	7	13	7	10	37
From 6 months onwards, cow’s milk can be given as an ingredient in homemade food	18	25	17	6	8
Potentially allergenic food (examples: eggs, fish, soy, seafood) can be given from the beginning of food introduction	8	14	7	16	29
Foods such as cornstarch biscuits and tapioca starch biscuits can be given from 9 months old	12	20	16	7	19
Acidic fruits such as pineapple, orange, pear, strawberry and kiwi should only be given after 9 months old	9	20	14	14	17
Soft foods (such as some fruits and vegetables) can be given in larger pieces for babies to take into their mouths from the beginning of food introduction	29	11	3	16	15
Ultra-processed foods should not be given to children until they are 2 years old	57	9	2	0	6

The average score in relation to knowledge about breastfeeding and complementary feeding was 17.6, with a minimum of 4 points and a maximum of 33. Fifty percent of professionals scored below 18 points, that is, they answered less than half of the questions correctly. Out of a total of 19 questions about knowledge about breastfeeding, the average score was 11.03, minimum of 3 and maximum of 17, with 68.9% getting 10 or more answers right. Physicians had an average of 12.11 right answers (95%CI 10.8; 13.2), nurses had 12.68 (95%CI 10.9; 14.2) and community health agents had 9.65 (95%CI 8.3; 11.0). The average score regarding knowledge about complementary foods, out of a total of 17 questions, was 6.54, with a minimum of 0 and a maximum of 17, with 77.0% totaling 9 points or less. The average score of physicians in this aspect was 7.44 (95%CI 5.7; 9.2), that of nurses, 8.05 (95%CI 6.0; 10.0), and that of community health agents, 5.32 (95%CI 4.4 ;6.3) de 5,32 (IC95% 4,4; 6,3). 

When the results were stratified into categories of right answers (≥15 right answers, 9-14 right answers, ≤8 right answers), in relation to breastfeeding, nurses showed greater knowledge on the topic, 7/19 with 15 or more right answers, and 2/18 of the physicians with the same score, while community health agents had less knowledge (15/37 with 8 or less right answers) (p-value 0.013). Two nurses had 15 or more right answers in relation to knowledge about food introduction, while physicians were more concentrated (9/18) between 9 and 14 right answers, followed by nurses (6/19) and community health agents (7/37). Community health agents were the category with the fewest right answers: 30 of the 37 agents had 8 or less right answers (p-value 0.019) (data not shown).

Of the 74 professionals, 35 partially agreed that they are qualified to provide information about breastfeeding, followed by 18 who strongly agreed. Physicians (8/18) and nurses (7/19) were the professionals who believed they were more qualified to provide such information when compared to community health agents (3/37) (p-value<0.001). Those who had studied the most (strongly agree and somewhat agree) about breastfeeding were physicians (8/18) followed by nurses (6/19). More than half of the community health agents (19/37) answered that they partially or strongly disagreed with this question (p-value 0.005), the same occurred regarding complementary feeding, but without a statistically significant difference (p-value 0.108) (Table 4).

**Table 4:** Attitudes of physicians,a nursesb and community health agentsc regarding the content of the food guide for Brazilian children under 2 years old (n=74). Botucatu, São Paulo, Brazil, 2023

Questions	Strongly agree	Somewhat agree	Neither agree or disagree	Somewhat disagree	Strongly disagree	p-value^d^
**I am qualified to provide information about breastfeeding**						0.001
Total	18	35	5	5	11	
Community health agent	3	14	5	4	11	
Nurse	7	11	0	1	0	
Physician	8	10	0	0	0	
**I need to study the topic of breastfeeding**						0,384
Total	38	29	4	1	2	
Community health agent	22	12	2	0	1	
Nurse	7	10	0	1	1	
Physician	9	7	2	0	0	
**I am qualified to provide information about complementary foods**						0.065
Total	7	34	9	11	13	
Community health agent	1	15	5	4	12	
Nurse	3	9	2	4	1	
Physician	3	10	2	3	0	
**I need to study the topic of complementary feeding**						0.179
Total	43	22	5	2	2	
Community health agent	25	8	3	0	1	
Nurse	9	7	0	2	1	
Physician	9	7	2	0	0	
**Have you studied about breastfeeding?**						0.005
Total	5	12	31	16	10	
Community health agent	1	2	15	10	9	
Nurse	3	3	11	2	0	
Physician	1	7	5	4	1	
**Have you studied about complementary foods?**						0.108
Total	5	9	24	20	16	
Community health agent	2	2	11	9	13	
Nurse	2	2	7	7	1	
Physician	1	5	6	4	2	

Note: an=18; bn=19; cn=37; dPearson’s chi-squared test.

As for practices regarding the content of the guide, 18 of the 74 professionals reported sometimes providing guidance on breastfeeding and 24 never did breastfeeding assessments during consultations and home visits. Nurses and physicians were the professionals who provided the most guidance on breastfeeding (8/19 and 6/18) and did breastfeeding assessment (8/19 and 3/18) when compared to community health agents (3/37 and 2/37) (p-value 0.016 and p-value 0.001). Regarding asking about and giving and guidance on feeding children aged 1 to 2 years, nurses (p-value 0.022), followed by physicians (p-value 0.011), were those who most frequently did so (Table 5).

**Table 5:** Practices of physicians,^a^ nurses^b^ and community health agents^c^ regarding the content of the food guide for Brazilian children under 2 years old (n=74). Botucatu, São Paulo, Brazil, 2023

Questions	Always	Often	Sometimes	Rarely	Never	p-value^d^
**Do you give guidance on breastfeeding?**						0.016
Total	17	17	18	11	11	
Community health agent	3	6	12	6	10	
Nurse	8	6	3	2	0	
Physician	6	5	3	3	1	
**Do you perform breastfeeding assessments during consultations and/or home visits?**						0.001
Total	13	14	11	12	24	
Community health agent	2	4	7	4	20	
Nurse	8	5	2	3	1	
Physician	3	5	2	5	3	
**Do you encourage the practice of breastfeeding when attending to mothers who have babies?**						0,424
Total	53	10	4	3	4	
Community health agent	24	5	3	1	4	
Nurse	14	4	0	1	0	
Physician	15	1	1	1	0	
**Do you ask postpartum women (mothers with less than 40 days after childbirth) about their mental health (fears, anguish, anxiety, etc.)?**						0.176
Total	38	16	13	3	4	
Community health agent	16	6	10	1	4	
Nurse	13	4	1	1	0	
Physician	9	6	2	1	0	
**Do you ask lactating women (women who breastfeed) if they smoke?**						0.756
Total	38	15	10	8	3	
Community health agent	17	7	5	5	3	
Nurse	11	3	3	2	0	
Physician	10	5	2	1	0	
**Do you ask lactating women (women who breastfeed) if they drink alcohol?**						0.453
Total	38	11	13	8	4	
Community health agent	15	6	8	4	4	
Nurse	12	2	2	3	0	
Physician	11	3	3	1	0	
**Do you give guidance on introducing complementary foods?**						0.131
Total	22	15	10	10	17	
Community health agent	7	7	4	5	14	
Nurse	8	4	4	3	0	
Physician	7	4	2	2	3	
**Do you ask about the feeding of children between 1 and 2 years old?**						0.022
Total	34	18	11	5	6	
Community health agent	12	10	10	3	2	
Nurse	13	4	1	1	0	
Physician	9	4	0	1	4	
**Do you give guidance on the feeding of children between 1 and 2 years old?**						0.011
Total	27	14	12	11	10	
Community health agent	6	8	10	6	7	
Nurse	13	2	1	3	0	
Physician	8	4	1	2	3	

Note: ^a^n=18; ^b^n=19; ^c^n=37; ^d^Pearson’s chi-squared test.

## Discussion

When assessing the knowledge, attitudes and practices of physicians, nurses and community health agents in primary health care in Botucatu, São Paulo, we found a high percentage of professionals with low knowledge about the content of the food guide for Brazilian children up to 2 years old, especially on complementary feeding. The attitudes and practices of these professionals showed important gaps, although nurses achieved better scores, followed by physicians. A possible explanation is that 69/74 had never had training on the guide. 

Greater knowledge about breastfeeding than about complementary feeding was found among Family Health Strategy teams in the city of Picos, in the state of Piauí ^12^. Shortcomings in knowledge about infant nutrition, especially food introduction, were identified among nurses from nine municipalities in the state of Paraíba ^13^.

This problem is not exclusive to Brazil. In several countries, conflicting knowledge about the duration of breastfeeding was found ^14^. Another systematic review regarding the competencies of health professionals as to breastfeeding beyond 12 months revealed supportive attitudes, but also passive or even hostile attitudes ^15^. These findings highlight the importance of health education actions. No similar international studies were found regarding complementary feeding, which indicates a gap in the literature.

The Food and Nutrition Surveillance System is an important tool for monitoring health professionals’ actions related to breastfeeding and complementary feeding in Primary Health Care in Brazil. However, coverage in Botucatu, where this study was conducted, is low, only having data on 5 children regarding exclusive breastfeeding and 11 regarding complementary feeding in 2022 ^16^. A cohort study carried out in Botucatu between 2015 and 2016, found a level of 62.7% breastfeeding at 6 months ^17^. Nationally, the Food and Nutrition Surveillance System indicated 54% exclusive breastfeeding and 44% of children between 6 and 23 months consuming ultra-processed foods in 2020 ^18^. There is evidence that breastfeeding and exclusive breastfeeding rates have been improving in Brazil, although still below recommended levels ^19^. These findings highlight the local data gap in this system and the importance of adequate guidance on child care.

Our study found statistical differences between professional categories, with community health workers in a poorer situation. This may be related to the fact that higher education is not mandatory for these professionals, this being a hypothesis that was also raised for the context found in Piauí ^9^. Community health agents in Uruburetama, Ceará, had insufficient knowledge about nutrition and had difficulties in providing guidance on breastfeeding and complementary feeding ^20^. The technical course for qualifying as a community health agent is currently mandatory and can be a strategy for reversing this reality, since professionals trained in this course have educational responsibilities, according to the National Primary Care Policy and the Community Health Agents Program ^21^ ^,^ ^22^.

Few professionals reported having knowledge of the guide, this being similar to the result found in Currais Novos, Rio Grande do Norte, in a study with 23 primary health care professionals, 14 of whom also reported not having knowledge of the guide (8). These findings are of concern, as professionals need to be trained to provide up-to-date information about breastfeeding and complementary feeding. This reinforces the need to popularize the food guide for children up to 2 years old, published in 2019.

Of note is the Brazilian Breastfeeding and Feeding Strategy. This is the Ministry of Health’s main program for training Primary Health Care professionals on breastfeeding and complementary feeding. Its participatory activities allow the exchange of experiences and adaptation to the local context ^23^. The need for effective implementation of this program is highlighted, as it is a powerful action for changing the scenario, in order to strengthen promotion, protection and support for breastfeeding and healthy eating in primary health care ^24^ ^,^ ^25^.

In Piracicaba, in the interior region of São Paulo state, professionals trained by the Brazilian Breastfeeding and Feeding Strategy obtained positive results in rates of exclusive breastfeeding, complementary breastfeeding and adequate food introduction. The results were also positive for reduced consumption of some ultra-processed foods, although there was an increase in the consumption of sweet food and candy ^26^. Following the implementation of the same strategy in Embu das Artes, also in São Paulo state, 37 workshops were held and 554 health professionals were trained. Several challenges were faced in implementing the action plans, which highlights the need to improve the training of professionals in breastfeeding and complementary feeding ^27^. 

Continuing education for health professionals, specifically focused on food and nutrition, identifying barriers and proposing solutions, is imperative for health care services, especially in Primary Health Care ^12^ ^,^ ^28^. Health education promotes integration between practice, communication and qualified listening, being essential for transforming habits and improving quality of life ^29^ ^,^ ^30^.

It should be noted that the Ministry of Health offers free training courses for Primary Health Care professionals through the UNA-SUS ^31^ and AVASUS ^32^ platforms. These courses cover topics such as breastfeeding, complementary feeding and the food guide itself. These courses are an opportunity for improving the results found and should be encouraged by Primary Health Care service managers.

We highlight that nutritionists are the most qualified professionals for working with food and nutrition actions, however, they are not part of the minimum primary care team. Nutritionists are part of multidisciplinary teams, a situation recently regulated, depending on adherence by municipal health departments ^33^. It is well known that the presence of nutritionists in health centers improves the health care of the population ^34^ ^-^ ^37^.

Some limitations of this study need to be considered, such as only including health professionals from a single municipality and a sample that represents approximately 30% of the health professional categories in question, although all of them were invited. There are two hypotheses for low participation in the study, with possible selection bias: firstly, work overload, which may have made professionals unwilling or led them to forget to take part, and lack of interest in the topic. Secondly, it is possible that only the most interested professionals participated, concealing a worse scenario of knowledge, attitudes and practices in relation to the food guide. Another limitation is the use of a non-validated questionnaire, prepared by the authors. Creating a validated questionnaire for this context is an opportunity for future investigations, enabling comparisons between studies and guaranteeing better methodological quality.

The knowledge of primary care physicians, nurses and community health agents about the food guide for Brazilian children up to 2 years old was deficient, as were their attitudes and practices. Health professionals working in the SUS, especially those who care for children under 2 years old, need to be aware of the guide and promote its content. Training these professionals with recommendations on breastfeeding and nutrition is essential for transforming the current health scenario and positively impacting future generations. We conclude that nutritionists are fundamental in this process, whether through the expansion of multidisciplinary teams or the inclusion of these professionals in health centers.

## Data Availability

The database and analysis codes used in the research are available upon request to the authors.
