# Supplementary material for: Knowledge, attitudes and practices of primary care professionals about the food guide for children up to 2 years old: a cross-sectional study, Botucatu, São Paulo, Brazil, 2023
Source: Epidemiol Serv Saude. 2024 Dec 13;33:e20240111. doi: 10.1590/S2237-96222024v33e20240111.en (PMC11654717; doi:10.1590/S2237-96222024v33e20240111.en)
Supplement: Translation

# Translation: Conhecimento, atitudes e práticas de profissionais da atenção primária sobre o guia alimentar para crianças até 2 anos: estudo transversal, Botucatu, São Paulo, 2023

**DOI:** 10.1590/S2237-96222024v33e20240111.pt

**Authors:** Vivian Lovison do Amaral, Giovana Canela Spadotto, Caroline de Barros Gomes

**Affiliations:** 1 Hospital das Clínicas da Faculdade de Medicina de Botucatu, Especialização em Redes de Atenção no Sistema Único de Saúde, Botucatu, SP, Brasil; 2 Universidade Estadual Paulista, Faculdade de Medicina de Botucatu, Programa de Pós-Graduação em Saúde Coletiva, Botucatu, SP, Brasil

**Keywords:** Aleitamento Materno, Nutrição do Lactente, Guias Alimentares, Atenção Primária à Saúde, Estudos Transversais

## Abstract

**Objetivo:**

Investigar conhecimento, atitudes e práticas de profissionais da atenção primária à saúde sobre aleitamento materno e alimentação complementar, de acordo com as recomendações do guia alimentar para crianças brasileiras menores de 2 anos.

**Métodos:**

Trata-se de estudo descritivo realizado de outubro a dezembro de 2023, com questionário *online* autoaplicável, direcionado a médicos, enfermeiros e agentes comunitários de saúde da atenção primária à saúde de Botucatu, São Paulo. Realizou-se análise descritiva e associação entre as categorias profissionais e o conhecimento, atitudes e práticas desses em relação ao conteúdo do guia, utilizando o teste qui-quadrado de Pearson.

**Resultados:**

Participaram 74 profissionais, sendo 37 agentes comunitários de saúde, 19 enfermeiros e 18 médicos. Metade dos profissionais apresentou baixo conhecimento acerca do conteúdo do guia, acertando menos da metade das questões, com destaque especial àquelas relacionadas à alimentação complementar, em que 57 profissionais acertaram menos da metade delas. Médicos e enfermeiros foram os profissionais que mais acreditaram estar mais capacitados para repassar informações relacionadas ao conteúdo do guia, quando comparados aos agentes comunitários de saúde (p-valor<0,001). Sobre as práticas, 18 profissionais alegaram realizar às vezes orientações sobre aleitamento materno, 24 alegaram nunca realizar avaliação da mamada durante as consultas e visitas domiciliares e 22 deles realizam orientações sobre introdução da alimentação complementar.

**Conclusão:**

O conhecimento de médicos, enfermeiros e agentes comunitários de saúde foi deficiente em relação ao conteúdo do guia. As atitudes e práticas relativas a tal conteúdo também foram comprometidas.

**Aspectos éticos**

A presente pesquisa respeitou os princípios éticos, obtendo os seguintes dados de aprovação

Comitê de ética em pesquisa: Faculdade de Medicina de Botucatu

Número do parecer: 6.463.135

Data de aprovação: 26/10/2023

Certificado de apresentação de apreciação ética: 74345323.2.0000.5411

Termo de consentimento: Obtido de todos os participantes antes da coleta

## Introdução

A alimentação nos primeiros dois anos de vida apresenta papel essencial para o crescimento, o desenvolvimento, a formação de hábitos e a saúde. Práticas alimentares inadequadas aumentam o risco de má-nutrição e doenças crônicas, considerando-se que o estado nutricional de crianças menores de dois anos é diretamente influenciado por essas práticas ^1^.

O primeiro alimento a ser ofertado ao bebê é o leite materno. Os benefícios deste, especialmente do aleitamento materno exclusivo até o sexto mês e o continuado até 2 anos ou mais, perpassam a mãe e vão até a fase adulta. São considerados como benefícios a diminuição do risco de câncer de ovário e mama, as menores taxas de diarreia e doenças respiratórias nos dois primeiros anos de vida e as menores taxas de obesidade na infância e na fase adulta ^2^. Após o período de aleitamento materno exclusivo, nos primeiros 180 dias de vida, a alimentação complementar apropriada se relaciona a melhores hábitos alimentares para toda a vida e menores taxas de alergias alimentares e de excesso de peso ^3^ ^,^ ^4^.

Cuidar da alimentação nessa faixa etária (0-23 meses) é essencial para o desenvolvimento adequado e a promoção da saúde ^5^. As orientações de profissionais da saúde devem ser atualizadas e baseadas em evidências científicas, pois condutas inadequadas durante esse período podem resultar em prejuízos à saúde ^6^.

A atenção primária à saúde é o principal campo de promoção da alimentação adequada nos primeiros dois anos de vida, sendo a porta de entrada do usuário no Sistema Único de Saúde (SUS), coordenando o cuidado. Os cuidados relativos à alimentação e nutrição nesse nível de assistência compreendem a promoção de uma alimentação adequada e saudável em todas as etapas da vida. Essa alimentação deve ser incentivada por todos os profissionais de saúde, respeitando seu núcleo de competências ^7^.

O guia alimentar para crianças brasileiras menores de 2 anos, publicado em 2019 pelo Ministério da Saúde, oferece diretrizes sobre aleitamento materno e alimentação complementar. Esse guia segue a recomendação da Organização Mundial da Saúde de que os governos desenvolvam diretrizes nacionais para apoiar as famílias e guiar políticas públicas ^1^. 

Em Currais Novos, no Rio Grande do Norte, evidenciou-se que elevado percentual de profissionais da atenção primária à saúde desconhecem e/ou têm dúvidas sobre aleitamento materno e alimentação complementar ^8^. Em seis unidades de Teresina, no Piauí, os profissionais possuíam conhecimento adequado sobre aleitamento materno exclusivo, no entanto precisavam melhorar suas orientações sobre alimentação complementar ^9^. Destaca-se que ambas as circunstâncias se referem ao aleitamento materno e à alimentação complementar, e não especificamente ao guia alimentar.

Ressalta-se a importância de que os profissionais de saúde conheçam as recomendações presentes no guia, e esse conhecimento, assim como a sua aplicação, seja investigado. O objetivo deste estudo foi investigar o conhecimento, as atitudes e as práticas de profissionais da atenção primária à saúde de Botucatu, São Paulo, sobre aleitamento materno e alimentação complementar, de acordo com as recomendações do guia alimentar para crianças brasileiras menores de 2 anos.

## Métodos

### Desenho e população de estudo

Trata-se de estudo descritivo realizado em Botucatu, município do Centro-Sul do estado de São Paulo. Botucatu apresentou, em 2022, população estimada de 145.155 habitantes. Em 2020, a taxa de mortalidade infantil foi 10,94 óbitos por 1 mil nascidos vivos. O último dado do Índice de Desenvolvimento Humano (0,800), de 2010, foi superior ao nacional ^10^.

A rede de atenção primária de Botucatu era composta por 22 unidades de saúde, sendo dois centros de saúde escola, seis unidades básicas de saúde e 14 unidades de saúde modelo Estratégia Saúde da Família ^11^.

Foram considerados elegíveis e convidados para o estudo todos os profissionais médicos, enfermeiros e agentes comunitários de saúde que atuassem em uma das 22 unidades de atenção primária à saúde do município, maiores de 18 anos. Considerando-se certa flutuação no número de profissionais, o município contava com, aproximadamente, 99 agentes comunitários de saúde, 72 enfermeiros e 85 médicos (dados não publicados). 

### Coleta de dados e variáveis

A coleta de dados ocorreu entre outubro e dezembro de 2023, por meio de um formulário *online* não validado, desenvolvido pelos autores. O convite aos profissionais foi feito via coordenação de cada unidade de saúde, após contato dos pesquisadores com a gestão municipal e a organização social que gerencia parte das unidades. A equipe realizou visitas presenciais, reforçando o convite com a gerência das unidades, e distribuiu lembretes com *QR code* do questionário aos profissionais elegíveis. Duas semanas após a visita, novo lembrete foi enviado via aplicativo de mensagem às gerências.

Antes do início do questionário, cada profissional realizou o aceite do Termo de Consentimento Livre e Esclarecido do estudo. O questionário contemplou questões de caracterização socioprofissional, seguindo para conhecimento, atitudes e práticas em relação ao aleitamento materno e alimentação complementar. Todas essas indagações constaram do conteúdo presente no guia alimentar para crianças brasileiras menores de 2 anos. 

Os itens relativos à caracterização do perfil e atuação profissional foram sexo biológico, idade, raça/cor da pele (branca, preta, parda, amarela, outra), profissão (agente comunitário de saúde, médico ou enfermeiro), unidade de saúde a que pertencia, tempo de atuação na atenção primária, escolaridade e formação acadêmica e profissional (graduação, especialização, residência, mestrado, doutorado). A seção seguinte do questionário abordou se o profissional conhecia o guia alimentar (sim, não), se já participou de algum treinamento sobre o guia (sim, não), treinamento sobre aleitamento materno (sim, não) e treinamento sobre alimentação complementar (sim, não), e há quanto tempo realizou esse treinamento. 

As seções sobre conhecimentos e atitudes referentes ao aleitamento materno e alimentação complementar contemplaram questões em escala Likert, na qual os participantes expressaram o seu grau de concordância por meio de cinco afirmações que variaram de “discordo totalmente” a “concordo totalmente”. Na seção sobre práticas, as questões envolveram as alternativas “nunca”, “raramente”, “ocasionalmente”, “frequentemente” e “sempre”. Os dados obtidos foram extraídos em planilha de Excel, na qual foi construído o banco de dados. Todas as questões eram de preenchimento obrigatório.

### Análise estatística

Foram realizadas análises descritivas com as variáveis investigadas, contemplando o cálculo de frequências absolutas e relativas para as variáveis categóricas e de média, intervalo de confiança de 95% e valores mínimos e máximos para as variáveis contínuas. A associação entre as diferentes categorias profissionais e os resultados encontrados sobre conhecer o guia, suas atitudes e práticas em relação ao conteúdo preconizado quanto ao aleitamento materno e à introdução da alimentação complementar foi realizada pelo teste qui-quadrado de Pearson. 

Na avaliação do conhecimento, as respostas dos profissionais foram categorizadas em certo ou errado, considerando-se certo a alternativa “concordo totalmente” ou “discordo totalmente”, a depender do questionamento feito, organizando-se essa variável como contínua. O número de acertos foi somado, separadamente para aleitamento materno (pontuação máxima=19) e alimentação complementar (pontuação máxima=18), e estratificado em três categorias: ≥15 acertos; 9-14 acertos; ≤8 acertos. A associação entre a categoria de acertos e a categoria profissional foi investigada por qui-quadrado de Pearson. Todas as análises foram realizadas no programa Statistical Package of Social Science for Windows, versão 20.0, considerando p-valor<0,05 como nível de significância estatística.

## Resultados

Participaram da pesquisa 74 profissionais de saúde, sendo 37 agentes comunitários de saúde, 19 enfermeiros e 18 médicos, o que perfaz, aproximadamente, 37% dos agentes comunitários de saúde, 26% dos enfermeiros e 21% dos médicos que atuam na atenção primária à saúde do município. A conclusão da graduação aconteceu há menos de cinco anos para 18 profissionais, e 31 deles alegaram ter feito especialização ou residência. Em relação ao modelo e tempo de atuação, 53/74 atuavam em unidade modelo Estratégia Saúde da Família e 40 alegaram estar atuando há menos de cinco anos na atenção primária à saúde (Tabela 1).

**Tabela 1:** Caracterização de médicos, enfermeiros e agentes comunitários de saúde atuantes na atenção primária à saúde e participantes do estudo. Botucatu, São Paulo, 2023 (n=74)

Características	n
**Idade (anos)**	
18-25	10
26-29	6
30-39	26
40-49	22
50-59	7
>60	3
**Sexo**	
Feminino	61
Masculino	13
**Raça/cor da pele**	
Branca	62
Parda	11
Preta	1
**Escolaridade**	
Ensino médio completo	14
Ensino superior completo	20
Ensino superior incompleto	10
Pós-graduação	30
**Profissão**	
Agente comunitário de saúde	37
Enfermeiro	19
Médico	18
**Tempo de conclusão da graduação (anos)** ^a^	
<5	18
5-9	8
10-14	6
15-20	8
>20	10
**Especialização/residência** ^a^	
Sim	31
Não	19
**Mestrado** ^a^	
Em curso	2
Concluído	10
Não cursou	38
**Doutorado** ^a^	
Em curso	1
Concluído	6
Não cursou	43
**Unidade de atuação**	
Estratégia Saúde da Família	53
Unidade Básica de Saúde	7
Centro de Saúde Escola	10
Policlínica	4
**Tempo de atuação na atenção primária à saúde (anos)**	
<5	40
5-9	7
10-15	6
15-20	11
>20	10

Nota: ^a^24 dados faltantes devido não terem cursado graduação.

Apenas 11 profissionais conheciam o guia e 19/74 não conheciam o documento. Foi observada diferença estatística entre as categorias profissionais, sendo os médicos e os enfermeiros os profissionais que mais conhecem o guia quando comparados aos agentes comunitários de saúde (p-valor 0,009). A maioria (n=46) discordou totalmente de que o guia é um material do Ministério da Saúde direcionado somente para os nutricionistas, e 34/74 concordaram totalmente que pode ser um material utilizado pelas famílias que possuem bebês nessa faixa etária, não havendo diferença estatística entre as categorias profissionais (p-valor 0,414 e p-valor 0,241). Do total de profissionais participantes, 69 não tiveram treinamento sobre o material, 42 alegaram ter realizado treinamento sobre aleitamento materno e 52 não realizaram treinamento sobre alimentação complementar, sem diferença estatística entre as categorias profissionais (dados não apresentados em tabelas).

A maioria dos profissionais (64/74) concordou totalmente que somente o leite materno é suficiente para o bebê nos seis primeiros meses de vida, e 69 deles discordaram totalmente de que mamas pequenas produzem pouco leite. A proporção que concordou totalmente que uma das posições de amamentar é deitada, junto ao bebê, foi apenas de 25/74. Questões como o consumo de canjica para aumento da produção de leite, a amamentação além dos 2 anos e a independência da criança e a mesma questão de amamentar deitada foram aquelas com mais dúvida entre os profissionais, reveladas por percentuais mais altos entre a concordância parcial e a discordância parcial (Tabela 2).

**Tabela 2:** Conhecimento de médicos, enfermeiros e agentes comunitários de saúde sobre o conteúdo de aleitamento materno do guia alimentar para crianças brasileiras menores de 2 anos. Botucatu, São Paulo, 2023 (n=74)

Questões	Concordo totalmente	Concordo parcialmente	Não concordo nem discordo	Discordo parcialmente	Discordo totalmente
Existe leite materno fraco	1	2	2	8	61
Mamas pequenas produzem pouco leite	0	1	0	4	69
Canjica é um exemplo de alimento que ajuda aumentar a produção do leite materno	5	10	24	9	26
É possível mães que tenham mamilo plano ou invertido amamentarem	48	15	5	3	3
É recomendado alternar as mamas em cada mamada	41	16	4	5	8
Mães infectadas pelo HIV podem amamentar	13	8	6	1	46
É indicada a amamentação na primeira hora de vida	64	5	4	0	1
A amamentação deve ocorrer sempre que a criança quiser, sem horários nem intervalos predefinidos	43	17	3	7	4
Somente o leite materno é suficiente para o bebê nos 6 primeiros meses de vida	64	8	0	0	2
O aleitamento materno deve ser exclusivo até os 6 meses (sem outros leites, fórmula, água, chá, sucos ou alimentos)	56	10	2	3	3
Amamentação para além de 2 anos prejudica o processo natural da criança de se tornar independente	8	12	12	15	27
Criança que mama no peito tem mais dificuldade para aceitar os outros alimentos	1	5	4	15	49
Uma das posições para a mãe amamentar é deitada, junto ao bebê	25	15	5	12	17
Mães com mamas ingurgitadas ("empedradas") devem ser orientadas a fazer compressa quente nas mamas	16	17	6	9	26
Chupeta pode causar confusão de bicos e atrapalhar o aleitamento materno	46	14	1	6	7
Mamadeira pode causar confusão de bicos e atrapalhar aleitamento materno	49	15	3	4	3
Lactantes (mulheres que amamentam) e ficam grávidas devem interromper a amamentação	12	9	8	12	33
Em dias muitos quentes, mesmo crianças em aleitamento materno exclusivo devem receber água	8	4	10	13	39
Mesmo com a introdução de novos alimentos, o aleitamento materno deve ser mantido até 2 anos ou mais	37	27	4	3	3

A maioria (55/74) dos profissionais concordou totalmente que a idade recomendada para o início da introdução alimentar é aos 6 meses de vida e que alimentos ultraprocessados não devem ser oferecidos à criança até ela completar 2 anos de idade (57/74; Tabela 3). Os profissionais que concordaram totalmente que alimentos com potencial alergênico como ovo, peixe, soja e frutos do mar podem ser oferecidos desde o começo da introdução alimentar foram somente 8. Mais da metade afirmou que suco de frutas pode ser oferecido desde o início da introdução alimentar (40/74). Foram observadas muitas dúvidas em relação aos demais questionamentos feitos, com elevados percentuais nas alternativas intermediárias (concordo parcialmente, não concordo nem discordo, discordo parcialmente).

**Tabela 3:** Conhecimento de médicos, enfermeiros e agentes comunitários de saúde sobre o conteúdo de alimentação complementar do guia alimentar para crianças brasileiras menores de 2 anos. Botucatu, São Paulo, 2023 (n=74)

Questões	Concordo totalmente	Concordo parcialmente	Não concordo nem discordo	Discordo parcialmente	Discordo totalmente
Os alimentos, desde o início da introdução alimentar, devem ser batidos no liquidificador ou *mixer* ou serem passados em uma peneira antes de serem ofertados para os bebês	19	8	7	7	33
Alho, cebola e cheiro verde podem ser incluídos para temperar a comida do bebê desde o primeiro dia em que ele começa a comer	22	17	11	4	20
Deve-se iniciar a introdução alimentar com as frutas e somente após boa aceitação destas introduzir a papinha/comidinha salgada	22	23	6	8	15
O grupo das leguminosas (feijões, lentilha, grão de bico, ervilha) deve compor a refeição desde o início da introdução da alimentação complementar para o bebê	28	17	9	11	9
No início da introdução alimentar, Não é recomendado oferecer carne de porco e ovo, mesmo que bem cozidos	29	17	6	9	13
As principais refeições (almoço e jantar) devem conter no pratinho um alimento de cada grupo alimentar: feijões; cereais ou tubérculos ou raízes; carnes ou ovos; legumes e verduras	46	13	10	3	2
Sucos de frutas (sem açúcar, somente com a fruta) podem ser oferecidos desde o início da introdução alimentar	40	10	3	7	14
A idade recomendada para o início da introdução alimentar são os seis meses de vida	55	14	3	1	1
Um dos sinais de prontidão para início da alimentação complementar é sentar-se sem nenhum ou com pouco apoio	24	16	15	6	13
Crianças não amamentadas e, que a família não possui recursos para comprar fórmula infantil, podem receber leite de vaca modificado até os 4 meses de idade	10	13	19	13	19
O açúcar, adicionado a preparações como bolos, sucos e tortas, pode ser oferecido à criança a partir do primeiro ano de vida	7	13	7	10	37
A partir de 6 meses, o leite de vaca pode ser oferecido como ingrediente de preparações caseiras	18	25	17	6	8
Alimentos com potencial alergênico (exemplos: ovo, peixe, soja, frutos do mar) podem ser oferecidos desde o começo da introdução alimentar	8	14	7	16	29
Alimentos como biscoito de maizena e biscoito de polvilho podem ser oferecidos a partir dos 9 meses de idade	12	20	16	7	19
Frutas ácidas como abacaxi, laranja, pera, morango e kiwi devem ser oferecidos somente após os 9 meses de idade	9	20	14	14	17
Alimentos macios (como algumas frutas e legumes) podem ser oferecidos em pedaços maiores para os bebês levarem até a boca desde o início da introdução alimentar	29	11	3	16	15
Alimentos ultraprocessados não devem ser oferecidos à criança até ela completar 2 anos de idade	57	9	2	0	6

A pontuação média em relação ao conhecimento sobre aleitamento materno e alimentação complementar foi 17,6, sendo a mínima de 4 e a máxima de 33 pontos. Cinquenta por cento dos profissionais ficaram abaixo de 18 pontos, ou seja, acertaram menos da metade das questões. No total de 19 questões sobre o conhecimento acerca do aleitamento materno, foi encontrada pontuação de 11,03, mínima de 3 e máxima de 17, sendo que 68,9% acertaram 10 ou mais questões. Os médicos apresentaram a média de 12,11 acertos (IC95% 10,8; 13,2), os enfermeiros, de 12,68 (IC95% 10,9; 14,2), e os agentes comunitários de saúde, de 9,65 (IC95% 8,3; 11,0). A pontuação média acerca do conhecimento sobre alimentação complementar, em um total de 17 questões, foi 6,54, com mínimo de 0 e máximo de 17, com 77,0% totalizando 9 ou menos pontos. A pontuação média dos médicos nesse quesito foi 7,44 (IC95% 5,7; 9,2), dos enfermeiros, de 8,05 (IC95% 6,0; 10,0), e dos agentes comunitários de saúde, de 5,32 (IC95% 4,4; 6,3). 

Quando estratificados os resultados em categorias de acertos (≥15 acertos, 9-14 acertos, ≤8 acertos), em relação ao aleitamento materno, os enfermeiros apresentaram maior conhecimento sobre o tema, 7/19 com 15 ou mais acertos, e 2/18 dos médicos com a mesma pontuação, sendo os agentes comunitários de saúde com menor conhecimento (15/37 com 8 ou menos acertos) (p-valor 0,013). Dois profissionais enfermeiros apresentaram 15 ou mais acertos em relação ao conhecimento sobre introdução alimentar, sendo os médicos mais concentrados (9/18) entre 9 e 14 acertos, seguidos de enfermeiros (6/19) e agentes comunitários de saúde (7/37). A categoria com menos acertos foi a dos agentes comunitários de saúde: 30 dos 37 agentes com 8 ou menos acertos (p-valor 0,019) (dados não apresentados em tabelas).

Concordaram parcialmente que estão capacitados para repassar informações sobre aleitamento materno 35 dos 74 profissionais, seguidos de 18 que concordaram totalmente. Os médicos (8/18) e os enfermeiros (7/19) foram os profissionais que acreditam estar mais capacitados para repassar tais informações quando comparados aos agentes comunitários de saúde (3/37) (p-valor<0,001). Quem mais tem estudado (concordo totalmente e concordo parcialmente) sobre aleitamento materno são os médicos (8/18), seguidos dos enfermeiros (6/19). Mais da metade dos agentes comunitários de saúde (19/37) responderam que discordavam parcialmente ou totalmente a este mesmo questionamento (p-valor 0,005), mesmo comportamento para alimentação complementar, mas sem diferença estatisticamente significante (p-valor 0,108) (Tabela 4).

**Tabela 4:** Atitudes de médicos,^a^ enfermeiros^b^ e agentes comunitários de saúde^c^ a respeito do conteúdo do guia alimentar para crianças brasileiras menores de 2 anos (n=74). Botucatu, São Paulo, 2023

Questões	Concordo totalmente	Concordo parcialmente	Não concordo nem discordo	Discordo parcialmente	Discordo totalmente	p-valor^d^
**Estou capacitado(a) para repassar informações sobre aleitamento materno**						0,001
Total	18	35	5	5	11	
Agente comunitário de saúde	3	14	5	4	11	
Enfermeiro	7	11	0	1	0	
Médico	8	10	0	0	0	
**Preciso estudar sobre a temática de aleitamento materno**						0,384
Total	38	29	4	1	2	
Agente comunitário de saúde	22	12	2	0	1	
Enfermeiro	7	10	0	1	1	
Médico	9	7	2	0	0	
**Estou capacitado(a) para repassar informações sobre alimentação complementar**						0,065
Total	7	34	9	11	13	
Agente comunitário de saúde	1	15	5	4	12	
Enfermeiro	3	9	2	4	1	
Médico	3	10	2	3	0	
**Preciso estudar sobre a temática de alimentação complementar**						0,179
Total	43	22	5	2	2	
Agente comunitário de saúde	25	8	3	0	1	
Enfermeiro	9	7	0	2	1	
Médico	9	7	2	0	0	
**Você tem estudado sobre aleitamento materno?**						0,005
Total	5	12	31	16	10	
Agente comunitário de saúde	1	2	15	10	9	
Enfermeiro	3	3	11	2	0	
Médico	1	7	5	4	1	
**Você tem estudado sobre alimentação complementar?**						0,108
Total	5	9	24	20	16	
Agente comunitário de saúde	2	2	11	9	13	
Enfermeiro	2	2	7	7	1	
Médico	1	5	6	4	2	

Nota: ^a^n=18; ^b^n=19; ^c^n=37; ^d^ Teste qui-quadrado de Pearson.

Sobre as práticas a respeito do conteúdo do guia, 18 dos 74 profissionais alegaram realizar às vezes orientações sobre aleitamento materno e 24 nunca realizar avaliação da mamada durante as consultas e visitas domiciliares. Foram os enfermeiros e os médicos os profissionais que mais realizam orientações sobre aleitamento materno (8/19 e 6/18) e avaliação de mamada (8/19 e 3/18) quando comparados aos agentes comunitários de saúde (3/37 e 2/37) (p-valor 0,016 e p-valor 0,001). Sobre o questionamento e a orientação sobre alimentação de crianças de 1 a 2 anos, são os profissionais enfermeiros (p-valor 0,022), seguidos dos médicos (p-valor 0,011), que mais frequentemente a realizam (Tabela 5).

**Tabela 5:** Práticas de médicos,^a^ enfermeiros^b^ e agentes comunitários de saúde^c^ a respeito do conteúdo do guia alimentar para crianças brasileiras menores de 2 anos (n=74). Botucatu, São Paulo, 2023

Questões	Sempre	Frequentemente	Às vezes	Raramente	Nunca	p-valor^d^
**Você realiza orientações sobre aleitamento materno?**						0,016
Total	17	17	18	11	11	
Agente comunitário de saúde	3	6	12	6	10	
Enfermeiro	8	6	3	2	0	
Médico	6	5	3	3	1	
**Você realiza avaliação de mamada durante consultas e/ou visitas domiciliares?**						0,001
Total	13	14	11	12	24	
Agente comunitário de saúde	2	4	7	4	20	
Enfermeiro	8	5	2	3	1	
Médico	3	5	2	5	3	
**Você incentiva a prática do aleitamento materno em seus atendimentos?**						0,424
Total	53	10	4	3	4	
Agente comunitário de saúde	24	5	3	1	4	
Enfermeiro	14	4	0	1	0	
Médico	15	1	1	1	0	
**Você questiona a puérpera (mãe com menos de 40 dias pós-parto) como está sua saúde mental (medos, angústias, ansiedade etc.)?**						0,176
Total	38	16	13	3	4	
Agente comunitário de saúde	16	6	10	1	4	
Enfermeiro	13	4	1	1	0	
Médico	9	6	2	1	0	
						
**Você pergunta para lactantes (mulheres que amamentam) se elas fumam?**						0,756
Total	38	15	10	8	3	
Agente comunitário de saúde	17	7	5	5	3	
Enfermeiro	11	3	3	2	0	
Médico	10	5	2	1	0	
**Você pergunta para lactantes (mulheres que amamentam) se elas ingerem bebida alcoólica?**						0,453
Total	38	11	13	8	4	
Agente comunitário de saúde	15	6	8	4	4	
Enfermeiro	12	2	2	3	0	
Médico	11	3	3	1	0	
**Você realiza orientações sobre introdução da alimentação complementar?**						0,131
Total	22	15	10	10	17	
Agente comunitário de saúde	7	7	4	5	14	
Enfermeiro	8	4	4	3	0	
Médico	7	4	2	2	3	
**Você pergunta sobre como está a alimentação de crianças que possuem entre 1 e 2 anos?**						0,022
Total	34	18	11	5	6	
Agente comunitário de saúde	12	10	10	3	2	
Enfermeiro	13	4	1	1	0	
Médico	9	4	0	1	4	
**Você orienta a alimentação de crianças que possuem entre 1 e 2 anos?**						0,011
Total	27	14	12	11	10	
Agente comunitário de saúde	6	8	10	6	7	
Enfermeiro	13	2	1	3	0	
Médico	8	4	1	2	3	

Nota: ^a^n=18; ^b^n=19; ^c^n=37; ^d^ Teste qui-quadrado de Pearson.

## Discussão

Ao avaliar o conhecimento, as atitudes e as práticas de médicos, enfermeiros e agentes comunitários de saúde da atenção primária à saúde de Botucatu, São Paulo, observou-se elevado percentual de profissionais com baixo conhecimento acerca do conteúdo do guia alimentar para crianças brasileiras menores de 2 anos, especialmente em alimentação complementar. As atitudes e as práticas desses profissionais apresentaram defasagens importantes, situação melhor entre enfermeiros, seguidos dos médicos. Uma possível explicação é que 69/74 nunca participaram de treinamentos sobre o guia. 

Observou-se maior conhecimento em aleitamento materno do que em alimentação complementar em equipes da Estratégia Saúde da Família de Picos, no Piauí ^12^. Foram identificadas deficiências no conhecimento sobre alimentação infantil, principalmente em introdução alimentar, entre enfermeiros de nove municípios da Paraíba ^13^.

Esse problema não é exclusivo do Brasil. Em diversos países, houve conhecimento conflitante sobre a duração da amamentação ^14^. Outra revisão sistemática em relação às competências de profissionais da saúde sobre aleitamento materno além dos 12 meses revelou atitudes de suporte, mas também posturas passivas ou até mesmo hostis ^15^. Esses achados destacam a importância de ações de educação em saúde. Não foram encontrados estudos internacionais semelhantes quanto à alimentação complementar, o que indica uma lacuna na literatura.

O Sistema de Vigilância Alimentar e Nutricional é uma ferramenta importante para monitorar as ações dos profissionais relacionadas a aleitamento materno e alimentação complementar na atenção primária à saúde. Porém, a cobertura em Botucatu, município do estudo, é baixa, com dados de 5 crianças sobre aleitamento materno exclusivo e 11 sobre alimentação complementar em 2022 ^16^. Em estudo de coorte realizado no município entre 2015 e 2016, observou-se 62,7% de aleitamento materno aos 6 meses ^17^. Nacionalmente, o Sistema de Vigilância Alimentar e Nutricional indicou 54% de aleitamento materno exclusivo e 44% de crianças entre 6 e 23 meses consumindo ultraprocessados em 2020 ^18^. Há evidências de que as taxas de aleitamento materno e aleitamento materno exclusivo vêm melhorando no país, mas aquém das recomendações ^19^. Esses achados destacam a lacuna de dados locais nesse sistema e a importância de orientações adequadas no cuidado infantil.

Neste estudo, houve diferenças estatísticas entre as categorias profissionais, com agentes comunitários de saúde em pior situação. Isso pode estar relacionado ao fato de que o nível superior não é obrigatório para esses profissionais, hipótese também levantada para o contexto no Piauí ^9^. Agentes comunitários de saúde em Uruburetama, no Ceará, apresentavam conhecimentos insuficientes sobre nutrição e dificuldades em orientar sobre aleitamento materno e alimentação complementar ^20^. O curso técnico em agente comunitário de saúde é atualmente obrigatório e pode ser uma estratégia para reverter essa realidade, uma vez que os profissionais formados nesse curso têm atribuições educativas, segundo a Política Nacional de Atenção Básica e o Programa de Agentes Comunitários de Saúde ^21^ ^,^ ^22^.

Poucos profissionais relataram conhecer o guia, resultado similar encontrado em Currais Novos, Rio Grande do Norte, com 23 profissionais, também da atenção primária à saúde, onde 14 profissionais deram o mesmo relato ^8^. Esses achados são preocupantes, pois os profissionais devem estar capacitados para repassar informações atualizadas sobre aleitamento materno e alimentação complementar. Isso reforça a importância da disseminação do guia alimentar para crianças menores de 2 anos, publicado em 2019.

Destaca-se a Estratégia Amamenta e Alimenta Brasil. Este é o principal programa do Ministério da Saúde para capacitação de profissionais da atenção primária à saúde sobre aleitamento materno e alimentação complementar. Suas atividades participativas permitem trocas de experiências e adaptação ao contexto local ^23^. Reforça-se a necessidade de implementação efetiva desse programa, sendo potente ação para a mudança do cenário, para fortalecer a promoção, a proteção e o apoio ao aleitamento materno e à alimentação saudável na atenção primária à saúde ^24^ ^,^ ^25^.

Em Piracicaba, interior de São Paulo, os profissionais capacitados pela Estratégia Amamenta e Alimenta Brasil obtiveram resultados positivos nos índices de aleitamento materno exclusivo, de aleitamento materno complementado e de introdução alimentar adequada. Os resultados foram positivos também para a redução no consumo de alguns alimentos ultraprocessados, contudo com aumento no consumo de doces e guloseimas ^26^. Devido à implementação desse mesmo programa em Embu das Artes, em São Paulo, 37 oficinas foram realizadas e 554 profissionais da saúde foram capacitados. Diversos desafios foram encontrados na implementação dos planos de ação, o que evidencia a necessidade de aprimorar a formação dos profissionais em aleitamento materno e alimentação complementar ^27^. 

A educação permanente dos profissionais de saúde, especificamente voltadas à alimentação e nutrição, identificando barreiras e propondo soluções, é um imperativo aos serviços de assistência à saúde, especialmente na atenção primária à saúde ^12^ ^,^ ^28^. A educação em saúde promove a integração entre prática, comunicação e escuta qualificada, sendo fundamental para transformar hábitos e melhorar a qualidade de vida ^29^ ^,^ ^30^.

É preciso pontuar que o Ministério da Saúde oferece cursos gratuitos de capacitação para profissionais da Atenção Primária à Saúde pelas plataformas UNA-SUS ^31^ e AVASUS ^32^. Tais cursos abordam temas como aleitamento materno, alimentação complementar e o próprio guia. Esses cursos são uma oportunidade para melhorar os resultados observados e devem ser incentivados pela gestão da atenção primária à saúde.

Destaca-se que o nutricionista é o profissional mais capacitado para atuar nas ações de alimentação e nutrição, contudo não faz parte da equipe mínima da atenção primária. Esse profissional integra equipes multiprofissionais, situação recentemente regulamentada, dependendo de adesão por parte dos municípios ^33^. É notório que a presença do nutricionista nas unidades de saúde melhora o cuidado com a saúde da população ^34^ ^-^ ^37^.

Algumas limitações precisam ser consideradas, como a inclusão de profissionais da saúde de um único município e uma amostra que representa aproximadamente 30% dos profissionais atuantes, embora todos tenham sido convidados. Existem duas hipóteses para a baixa participação, com possível viés de seleção: a sobrecarga de trabalho, que pode ter feito com que os profissionais não quisessem ou esquecessem de participar, e a falta de interesse no tema. Na segunda hipótese, é possível que apenas os profissionais mais interessados tenham participado, ocultando um cenário pior de conhecimento, atitudes e práticas em relação ao guia. Outra limitação é o uso de questionário não validado, elaborado pelos autores. A criação de questionário validado para esse contexto representa uma oportunidade para futuras investigações, o que permite comparações entre estudos e garante melhor qualidade metodológica.

O conhecimento de médicos, enfermeiros e agentes comunitários de saúde da atenção primária sobre o guia alimentar para crianças brasileiras menores de 2 anos foi deficiente, assim como suas atitudes e práticas. É necessário que os profissionais da saúde atuantes no SUS, especialmente os que assistem à população de menores de 2 anos, conheçam o guia e promovam seu conteúdo. Capacitar esses profissionais com as recomendações sobre aleitamento materno e alimentação é essencial para transformar o atual cenário de saúde e impactar positivamente as futuras gerações. Conclui-se que o nutricionista é fundamental nesse processo, seja por meio da expansão das equipes multiprofissionais, seja pela inclusão desse profissional nas unidades de saúde.

## Data Availability

The database and analysis codes used in the research are available upon request to the authors.
